# A function blocking anti-mouse integrin α5β1 antibody inhibits angiogenesis and impedes tumor growth *in vivo*

**DOI:** 10.1186/1479-5876-5-61

**Published:** 2007-11-27

**Authors:** Vinay Bhaskar, Dong Zhang, Melvin Fox, Pui Seto, Melanie HL Wong, Pauline E Wales, David Powers, Debra T Chao, Robert B DuBridge, Vanitha Ramakrishnan

**Affiliations:** 1 Department of Research, PDL Biopharma, Inc., Fremont, CA 94555, USA

## Abstract

**Background:**

Integrins are important adhesion molecules that regulate tumor and endothelial cell survival, proliferation and migration. The integrin α5β1 has been shown to play a critical role during angiogenesis. An inhibitor of this integrin, volociximab (M200), inhibits endothelial cell growth and movement *in vitro*, independent of the growth factor milieu, and inhibits tumor growth *in vivo *in the rabbit VX2 carcinoma model. Although volociximab has already been tested in open label, pilot phase II clinical trials in melanoma, pancreatic and renal cell cancer, evaluation of the mechanism of action of volociximab has been limited because this antibody does not cross-react with murine α5β1, precluding its use in standard mouse xenograft models.

**Methods:**

We generated a panel of rat-anti-mouse α5β1 antibodies, with the intent of identifying an antibody that recapitulated the properties of volociximab. Hybridoma clones were screened for analogous function to volociximab, including specificity for α5β1 heterodimer and blocking of integrin binding to fibronectin. A subset of antibodies that met these criteria were further characterized for their capacities to bind to mouse endothelial cells, inhibit cell migration and block angiogenesis *in vitro*. One antibody that encompassed all of these attributes, 339.1, was selected from this panel and tested in xenograft models.

**Results:**

A panel of antibodies was characterized for specificity and potency. The affinity of antibody 339.1 for mouse integrin α5β1 was determined to be 0.59 nM, as measured by BIAcore. This antibody does not significantly cross-react with human integrin, however 339.1 inhibits murine endothelial cell migration and tube formation and elicits cell death in these cells (EC_50 _= 5.3 nM). In multiple xenograft models, 339.1 inhibited the growth of established tumors by 40–60% (*p *< 0.05) and this inhibition correlates with a concomitant decrease in vessel density.

**Conclusion:**

The results herein demonstrate that 339.1, like volociximab, exhibits potent anti-α5β1 activity and confirms that inhibition of integrin α5β1 impedes angiogenesis and slows tumor growth *in vivo*.

## Background

Angiogenesis is the process by which nascent blood vessels form from existing vasculature to supply new tissue with nutrients. This process was proposed by Folkman over three decades ago to drive tumor growth beyond a few millimeters [1,2]. Since that time, numerous angiogenesis inhibitors have been shown to inhibit vessel growth in models of neovascularization and restrict the growth of tumors in pre-clinical models of cancer. Currently, anti-angiogenic agents that are approved for the treatment of cancer include sunitinib [3,4], sorafenib [5,6] and bevacizumab [7,8], and many others are in late stage clinical testing.

Tumors secrete multiple growth factors that drive activation, migration and proliferation of vascular endothelial cells (EC's), including TGF-α, bFGF and VEGF [9]. These agents bind their respective receptors on EC's to initiate signaling cascades that culminate in pro-angiogenic events. To date, the majority of angiogenesis inhibitors have focused on disrupting growth factor signalling, including the aforementioned clinically approved agents, which primarily target the VEGF axis [4,5,8]. However, growth factors are not the only pro-angiogenic molecules that influence the tumor microenvironment. For example, tumor cells and tumor-associated macrophages are known to secrete matrix metalloproteinases, such as MMP-9 and MMP-2 [10]. These enzymes degrade the basement membrane, exposing components of the extracellular matrix, including fibronectin. These exposed ECM proteins drive angiogenesis by ligating integrins, which play a central role in the angiogenic program [11-14].

Integrins are heterodimeric signalling and adhesion molecules consisting of an alpha chain and a beta chain. Ligands for these receptors bind integrins and induce EC shape change, motility and growth [12,14]. One well-characterized example of an integrin-ECM interaction is that between fibronectin and integrin α5β1, an integrin that is up-regulated in proliferating EC's [15,16]. Ligation of α5β1 has been shown to promote cell survival through Bcl-2, migration through RhoA and proliferation through ERK, Akt and FAK-dependent mechanisms [13,15-20]. Activation of these and other cellular programs through integrin α5β1 in endothelial cells results in angiogenesis. Conversely, blockade of α5β1 ligation has been demonstrated to inhibit angiogenesis, at least in part through the inhibition of signalling and the induction of the cell death program through caspases [15-21].

Volociximab is a chimeric, function blocking antibody that targets integrin α5β1. We have previously shown that this antibody elicits cell death in dividing endothelial cells, inhibits angiogenesis in a cynomolgus monkey model of choroidal neovascularization [21] and slows tumor growth in a rabbit VX2 carcinoma model [22]. Volociximab, however, does not cross-react with rodent α5β1, precluding its use in standard mouse xenograft models of cancer. Although commercially available antibodies against mouse α5 exist, we and others have found that these monoclonals do not elicit cell death or inhibit tumor progression *in vivo *[23,24].

To determine whether blockade of α5β1 in murine disease models results in inhibition of angiogenesis and tumor growth, we sought to generate a function blocking antibody that closely mirrored the known properties of volociximab. To this end, we generated a panel of rat-anti-mouse integrin α5β1 antibodies and subjected them to a rigorous screening strategy designed to identify antibodies that reproduce the known properties of volociximab that are associated with its efficacy *in vitro *and *in vivo*.

## Methods

### Purification of mouse placental α5β1

Mouse placental integrin α5β1 was purified by affinity chromotagraphy using rat anti-mouse α5β1 antibody BMC5 (Chemicon) essentially as described [25]. Briefly, antibody was coupled to CNBr-coupled Sepharose (Pharmacia) and mouse placenta was homogenized using a Polytron tissue homogenizer in lysis buffer [200 mM octyl-β-gluocopyranoside, 1 mM phenylmethylsulfonyl fluoride, 1 mM MgCl_2_, 0.5 mM CaCl_2_, 150 mM NaCl, 25 mM HEPES (pH 7.0)], followed by incubation on ice. After centrifugation, the cleared lysate was loaded onto a BMC5-Sepharose column and washed with 20 column volumes of lysis buffer with the octyl-β-gluocopyranoside substituted with 0.1% Nonidet P-40. Integrin was eluted at low pH in a buffer containing 10 mM EDTA and dialyzed against phosphate buffered saline.

### Cloning of mouse integrin and purification of α5β1-Fc fusion protein

Total RNA (1 μg) isolated from mouse tissue was solubilized in Trizol™ (Invitrogen) and cDNA was produced using SuperScript II (Invitrogen). Full-length α5 and β1 were amplified by PCR using gene-specific primers (Qiagen N.V.). PCR products were subcloned into the pCR4 TOPO vector (Invitrogen) and verified by sequencing. To generate the α5 and β1-Fc fusion genes, regions encoding the extracellular domains of the α5 and β1 integrin genes were isolated by restriction digest and individually subcloned into the expression vector DEF38 (ICOS) to create an in-frame fusion between each of the extracellular domains and the constant region of the human γ1 immunoglobulin heavy chain gene. A stable cell line was generated by co-transfecting 293 cells with a 50:50 mix of α5-Fc and β1-Fc plasmid DNAs using FuGene (Roche). Conditioned medium was harvested and Fc fusion protein was purified by standard methods using Protein G Sepharose.

### Generation of anti-mouse integrin α5β1 antibodies

Female Sprague-Dawley rats (Simonsen) were immunized intraperitoneally with α5β1 purified from mouse placenta or with a mouse α5β1-Fc fusion protein. Monoclonal antibodies were generated by standard techniques fusing spleen cells from immunized mice with an NSO-derived fusion partner (American Type Culture Collection). A panel of α5β1-specific antibodies was identified by ELISA using the Fc-fusion protein and by flow cytometry analysis of binding to mouse endothelial cells and cell lines.

### Integrin α5β1 binding ELISA

Purified α5β1-Fc (100 ng) was plated into wells in 50 mM Na_2_CO_3 _(pH 8.6), 0.5 mM each of CaCl_2_, MgCl_2_, and MnCl_2 _at 4°C overnight. Plates were blocked with 5% BSA in PBS for 1 hr with shaking. Following washes in PBS with 0.05% Tween-20, wells were incubated for 1 hr with serial dilutions of candidate antibodies or control rat IgG (Jackson ImmunoResearch) in 1% BSA in PBS with 0.05% Tween-20. Wells were then washed and incubated with 100 μL/well of the goat anti-rat IgG-HRP (1:5,000) for 1 hr. Washed plates were incubated with TMB substrate (Sigma), developed with 650 Stop Reagent (BioFX), and read at A_650_.

### Fibronectin binding inhibition ELISA

Cellular mouse fibronectin-coated plates (250 ng in coating buffer overnight at 4°C) were washed with PBS containing 0.05% Tween-20, and blocked as described above. Plates were incubated with α5β1-Fc fusion protein in the presence of various concentrations of the indicated antibodies for 1 hr at room temperature. Following 2 washes, plates were further incubated with goat anti-human IgG Fc-HRP (1:5000; Jackson ImmunoResearch) at room temperature for 1 hr. Plates were then washed 5 times and incubated with TMB substrate, developed, and read at A_650_.

### Flow cytometry

Cells were removed with 20 mM EDTA in Tris-HCl (pH 8.0) and blocked by centrifugation in HBSS containing 3% heat-inactivated FBS, 1% normal goat serum (Sigma) and 1% BSA at 4°C for 5 min. Cells were incubated for 1 hr at 4°C with the indicated supernatant or antibody (10 μg/mL) in FACS buffer (PBS containing 0.1% BSA). Excess mAb was removed by centrifugation and cells were resuspended in FACS buffer containing anti-rat IgG-PE secondary antibody (Southern Biotech). After an additional wash, fluorescence intensity was measured on a FACSCalibur flow cytometer (Becton Dickinson).

### Migration assay

BD HTS FluoroBlok 96-well plates (top plate) were coated with mouse plasma fibronectin (10 μg/ml; Upstate) in PBS and air-dried. 200 μl migration medium (MM; RPMI + 0.1% BSA) with or without antibody was dispensed into a BD Falcon 96-Square Well Flat Bottom Assay plate (bottom plates) and 10,000 cells/well in 50 μl was added to the top plate in MM. After lowering the top plate into the lower plate, cells were incubated at 37°C at 5% CO_2 _for 4 hr. Cells were stained with 2 μM Calcien-AM (Invitrogen) in MM and visualized using the Discovery-1 High Content Screening System (Molecular Devices).

### Surface plasmon resonance

Affinities between α5β1-Fc and anti-integrin antibodies were analyzed using a BIAcore 3000 essentially as described [26,27]. Goat anti-rat Fc antibodies were immobilized on a Research Grade CM5 chip using an amine coupling kit (BIAcore). Rat-anti-mouse α5β1 antibodies were captured onto goat anti-rat Fc surfaces, followed by injection of mouse α5β1 in running buffer [(10 mM HBS, 2 mM CaCl_2_, 1 mM MnCl_2_, 700 mM NaCl (pH 7.4)] at a flow rate of 30 μL/min at 25°C. Association phase occurred over 3 min and dissociation over 1.5 hr. Kinetics of binding was calculated from data at 6 different concentrations of analyte (512 nM, 128 nM, 32 nM, 8 nM, 2 nM, 0.5 nM), using the BIAevaluation program. Each goat anti-rat Fc surface was regenerated at the end of each cycle by a quick injection of 30 mM HCl. Double-referencing was applied to eliminate responses from the reference surface and buffer-only control. Affinity constant (K_D_) was obtained by simultaneously fitting the association and dissociation phases of the sensorgram from the analyte concentration series using the 1:1 Langmuir model from the BIAevaluate software.

### Annexin V cell death assay

Primary mouse endothelial cells were incubated with anti-mouse α5β1 antibodies at 10 μg/ml for 16 hr, after which cell membrane phosphatidylserine was detected using Oregon Green 488 conjugated Annexin V. Harvested cells were washed with Annexin binding buffer [(ABB; 10 mM HEPES, 140 mM NaCl, 2.5 mM CaCl_2 _(pH 7.4]) and incubated in ABB (100 μl) containing Annexin V (10 μl). Following washing, cells were suspended in ABB containing 0.5 μg/mL propidium iodide (200 μl) and assessed by flow cytometry.

### HUVEC and murine EC *in vitro *angiogenesis models

HUVEC or murine lung EC's (5 × 10^5 ^cells/ml) were seeded in Hank's balanced salt solution (HBSS) containing 3 mg/ml fibrinogen and 200 μg/ml aprotinin (Roche) at 37°C, as described previously [21]. For each condition, EC/fibrinogen/aprotinin mixture (500 μL) was quickly transferred to a 24-well tissue culture plate containing α-thrombin (1 U) and gently mixed. The resulting fibrin matrices were polymerized at 37°C for 20 min. Antibody was added in Medium 200 [1 ml, without LSGS and containing 20% human or mouse serum (Rockland Immunochemicals), and rhTGF-α (0.01 μg/ml), rhHGF (0.1 μg/ml) and rhVEGF and/or rmVEGF (0.1 μg/ml)]. Tubes formed over 6 days, with one medium change (Medium 200 plus serum only) at day 3. For visualization of tubes, matrices were fixed with 4% formaldehyde for 4 hr and stained overnight with 1 ml phalloidin-Alexa 488 (1.75 U/ml) in 50% fetal calf serum (FCS) containing 0.25% saponin. Vessel formation was visualized using the Discovery-1 High Content Screening System (Molecular Devices) and quantified using the accompanying software.

### Immunohistochemistry

Xenograft tumors derived from cells inoculated subcutaneously in ICR-SCID or SCID-Beige mice (Taconic Farms) were frozen in OCT compound and stored at -70°C. Cryostat tissue sections (4-5 μm) were fixed in acetone for 10 min, air dried and incubated in 0.03% H2O2 for 10 min. After successive incubations with Avidin block, Biotin block and Protein block solutions for 15 min each, samples were treated with the indicated antibodies or biotin-conjugated rat anti-mouse CD31 mAb MEC 13.3 or biotin-conjugated IgG2a isotype control (2.5 μg/ml; BD Pharmingen) for 60 min. Sections were developed using the Vectastain Elite ABC kit (Vector Laboratories) and stable diaminobenzidine (Dako). All staining procedures were performed using a Dako Autostainer at room temperature.

### *In vivo *xenograft studies

Six- to eight-week-old ICR SCID or SCID-Beige female mice, obtained from Taconic Farms and maintained in micro-isolator cages, received subcutaneous injections on the right flank of 5 × 10^6 ^A673 human rhabdomyosarcoma or 1 × 10^7 ^SVR murine angiosarcoma cells. Tumors were allowed to establish for 7–18 days, reaching an average of 50–100 mm^3^, as determined by caliper measurement. Animals were distributed into groups of ten and received vehicle (PBS) or rat anti-mouse integrin α5β1 antibody (200 μl at 1.0 mg/ml). Reagents were delivered by intraperatoneal injection twice or thrice weekly for the duration of the studies. Tumor volume was measured twice weekly, and clinical and mortality observations were performed daily according to Institutional Animal Care and Use Committee regulations.

## Results

### Generation and characterization of rat anti-mouse integrin α5β1 antibodies

To generate antibodies directed against murine integrin α5β1, Sprague-Dawley rats were immunized with mouse α5β1-Fc fusion protein or with affinity-purified integrin from mouse placenta. Supernatants from the resulting hybridomas were subjected to a number of assays designed to identify clones that produced antibodies encompassing characteristics that define volociximab, including heterodimer specificity, blocking activity, high affinity binding to the Fc fusion protein and binding to endothelial cells [21]. Over sixty clones were screened for these functional properties.

**Figure 1:**
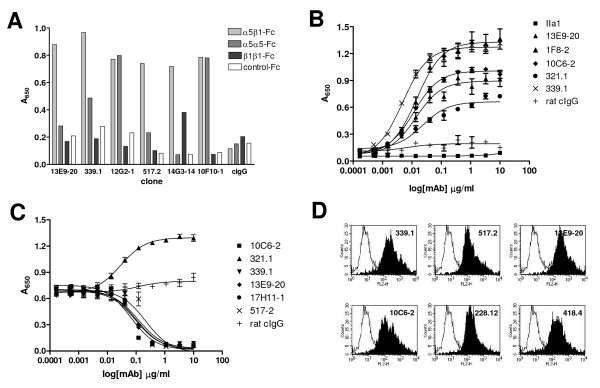
**Representative hybridoma screening results**. Hybridoma supernatants were compared by ELISA for binding to murine α5β1-Fc heterodimer, α5-Fc or β1-Fc homodimer or an irrelevant Fc fusion protein, (A). Hybridomas were found to produce antibodies with specific preferences for each protein. Supernatants were further tested for binding to murine EC's by flow cytometry, (B). The majority of antibodies tested recognized cell surface integrin. Antibodies purified from supernatants that recognized murine EC's by flow cytometry and showed a preference for α5β1 heterodimer were compared for their capacity to inhibit the binding of α5β1-Fc to mouse fibronectin by competitive ELISA, (C). Many antibodies inhibited integrin-fibronectin binding; one antibody, 321.1, was found to promote binding. Antibodies were assessed for binding to cellular integrin α5β1 on SVR cells by flow cytometry, (D). Most of the antibodies that bound integrin α5β1 by ELISA also bind cell surface integrin *in vitro*.

Selected antibodies represented a range of binding characteristics. These included antibodies specific for α5, β1 or α5β1 heterodimer, as determined by ELISA (Figure 1A), and spanned a range of relative affinities (Figure 1B) and abilities to block binding of α5β1 to fibronectin (Figure 1C). Interestingly, one antibody, 321.1, increased binding of integrin to fibronectin, suggesting that it may recognize a ligand-induced binding site (LIBS). In general, most α5-, β1- and α5β1 heterodimer-specific antibodies recognized integrin α5β1 on immortalized endothelial cells (Figure 1D). Taken together these assays identified a number of antibodies that resemble volociximab *in vitro*.

### Cross-reactivity assays

**Figure 2:**
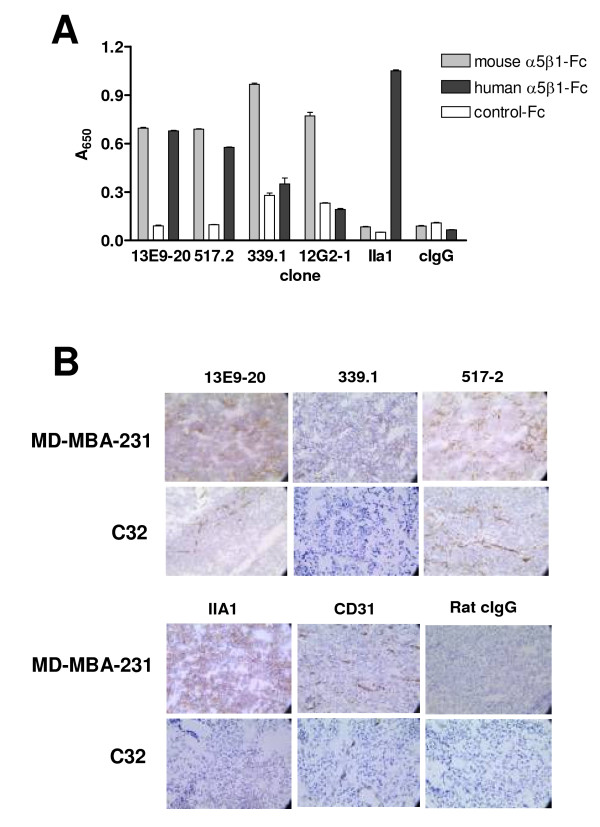
**Testing antibody cross-reactivity to human α5β1**. Representative antibodies were compared by ELISA for binding to human and murine α5β1-Fc or irrelevant Fc fusion proteins, (A). A subset of competitive antibodies cross-reacted with human integrin. Antibodies were tested by immunohistochemistry for staining of sections from C32 melanoma (α5β1 negative) or MDA-MB-231 breast carcinoma (α5β1 positive) xenografts, (B). The majority of antibodies tested stained murine α5β1 on tumor vasculature, but only antibodies found to cross-react with human α5β1 by ELISA specifically stained MDA-MB-231 xenograft cells as well. IIA1, the mouse parent antibody of volociximab, which recognizes only human integrin, anti-mouse CD31, which stains mouse vessels, and pooled rat IgG are shown as controls.

Volociximab does not bind to murine α5β1 [22], suggesting that differences exist in this critical functional epitope between mice and humans. A number of antibodies were cross-reactive for human integrin, including a majority of the α5 and heterodimer specific antibodies and all of those tested that bound β1 (Figure 2A). Immunohistochemical analysis of sections from human α5β1 positive (MDA-MB-231) and negative (C32) xenograft tumors further confirmed that antibodies that cross-reacted to human integrin by ELISA also did so on tissue sections (Figure 2B). For example, 517-2, which cross-reacts by ELISA, binds to tumor cells in the α5β1 positive MDA-MB-231 xenograft, and not to C32 xenograft tumor cells, but binds to murine α5β1 on the vasculature and stroma of both xenografts. Antibody 339.1, on the other hand, does not cross-react to human integrin by ELISA and does not bind tumor cells in either the MDA-MB-231 or the C32 xenograft, yet it recognizes murine α5β1 on vessels and stroma. The panel of antibodies, therefore, represents antibodies that bind human α5β1 and those that do not.

### Migration assays

**Figure 3:**
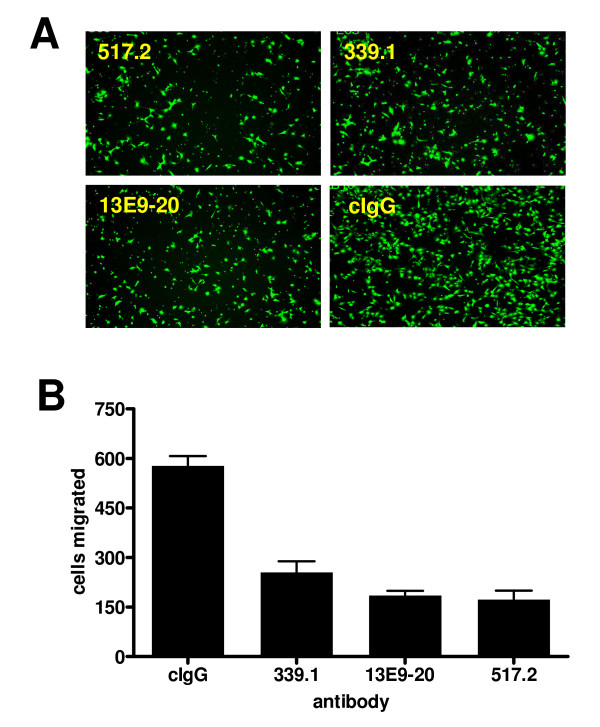
**Cell migration assays**. Immortalized murine endothelial SVEC-4 cells, (A), were assessed for chemotaxis to fibronectin coated on the opposite side of transwell membranes in the presence or absence of anti-integrin antibodies. Cells were stained with Calcein AM and visualized by fluorescence microscopy. Migration in quintiplicate wells was quantified using the Discovery-1 system, (B). Antibodies that inhibited binding of α5β1 binding to fibronectin also inhibited migration towards fibronectin.

High affinity, function-blocking antibodies against integrin α5β1 have been reported to inhibit endothelial cell migration and tube formation and elicit cell death *in vitro *[15,16,21]. A subset of antibodies from the panel was assessed for these effects in murine cell-based assays. Inhibition of migration towards fibronectin was determined using the murine hemangioma line SVEC. Of the antibodies tested, 13E9-20, 517-2 and 339.1 were among the most potent (Figure 3A, B), each inhibiting migration by greater than 50% relative to an IgG control. The extent of inhibition for each antibody was statistically significant (*p *< 0.05) relative to control.

### BIAcore analysis

Volociximab has a very high affinity for human α5β1 [21]. We were therefore interested in identifying antibodies with similarly high affinity for mouse α5β1. Selected antibodies were purified from supernatants using Protein A affinity chromatography and assessed for binding to mouse and human α5β1 by BIAcore. Of the human cross-reactive set, 517-2 had the highest affinity (K_D _= 0.21 nM) and of the non-cross-reactive set, antibody 339.1 bound tightest (K_D _= 0.59 nM). The affinities of both antibodies were sub-nanomolar, comparing favorably with volociximab (K_D _= 0.32 nM).

### Cell death assays

**Figure 4:**
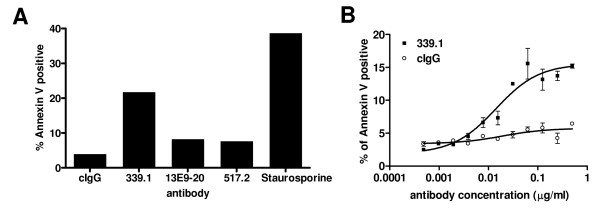
**Annexin V cell death assays**. Primary mouse endothelial cells were incubated with 339.1, 517-2 or 13E9-20 (10 μg/ml) for 16 hours and assessed for Annexin V-Alexa Fluor 488 binding by flow cytometry. Cells were counterstained with propidium iodide (PI) to follow non-specific death and the percentage of cells that stained positive for Annexin V and negative for PI was plotted. Representative results from three individual experiments are shown, (A). Only antibody 339.1, which does not cross-react with human α5β1, elicited cell death in these cells. A dose response curve for 339.1 in this assay using SVR murine angiosarcoma cells revealed an EC_50 _of 5.3 nM, (A).

The highest affinity antibodies that inhibited α5β1 binding to fibronectin and cell migration, were human cross-reactive antibody 517-2 and mouse-specific antibody 339.1. These two antibodies, and a third, lower affinity antibody (K_D _= 11.5 nM) that binds both mouse and human integrin, 13E9-20, were assessed for their ability to elicit cell death in mouse ECs in a manner analogous to that of volociximab on HUVEC. Primary murine endothelial cells were incubated with the antibodies for 16 hours and Annexin V binding was assessed by flow cytometry. 339.1 induced significant apoptosis in these cells whereas 517-2 and 13E9-20 did not, despite 517-2 and 339.1 having similar affinities for mouse integrin (Figure 4A). 339.1 also induced cell death in the T-antigen transformed murine endothelial cell line SVR, with an EC_50 _of 5.3 nM (Figure 4B), compared to an EC_50 _of 2.5 nM for volociximab on HUVEC. This finding suggests that although 339.1 and 517-2 have similar biological capabilities and similar affinities, initiation of the cell death program requires binding to a highly specific epitope, specifically recognized by 339.1.

### Tube formation assays

**Figure 5:**
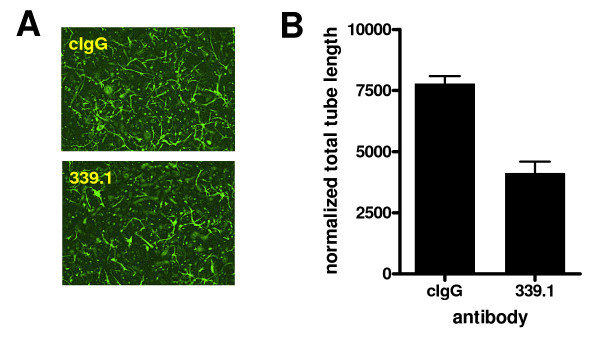
***In vitro *angiogenesis assays**. Primary endothelial cells isolated from mouse lung were induced to form tubes in a three-dimensional fibrin matrix under the influence of growth factors, in the presence of 339.1 or control IgG, (A). Results from triplicate wells were quantified using the Discovery-1 system (B). Inclusion of 339.1 in this assay results in a decrease in tube formation.

To determine if 339.1 also inhibited the formation of capillary-like structures in an *in vitro *angiogenesis assay, endothelial cells isolated from mouse lungs were induced to form tubes in a fibrin matrix in the presence or absence of antibody. Antibody 339.1 significantly inhibited tube formation (Figure 5A, B; *p *= 0.018), although this activity was not as robust as the inhibitory effects exerted by the human cross-reactive antibodies or volociximab on HUVEC tube formation (data not shown; [21]). This suggests that murine lung microvascular cells are less sensitive, in general, to anti-α5β1 activity, relative to human umbilical vein endothelial cells, in this assay. Nevertheless, these results demonstrate that antibodies in this panel can inhibit the ability of mouse and human endothelial cells to adopt a vessel-like structure in a three dimensional assay.

### Xenograft experiments

**Figure 6:**
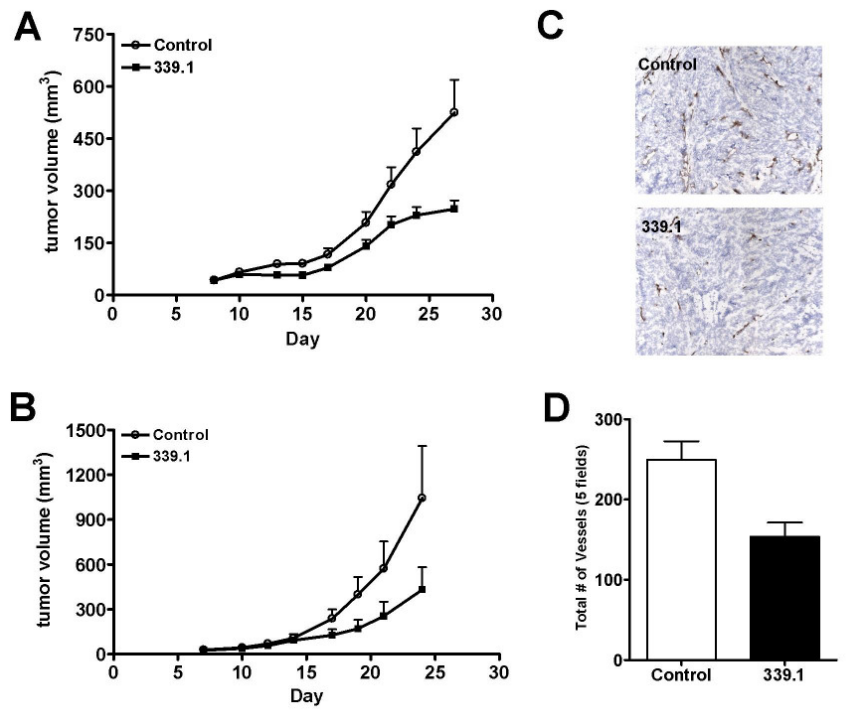
**339.1 inhibits angiogenesis and tumor growth *in vivo***. Mice bearing established SVR murine angiosarcoma, (A) or A673 human rhabdomyosarcoma tumors, (B), were treated with 339.1 (10 mg/kg, intraperitoneally, thrice or twice weekly, respectively) or vehicle control and tumor volume was monitored using vernier calipers. 339.1 inhibits tumor growth *in vivo *relative to control. Results were statistically significant in both settings. A673 tumors were resected from 339.1- and control-treated mice and frozen sections were assessed for vessel density by immunohistochemical staining for CD31, (C). Vessel density was significantly reduced in tumors from animals treated with 339.1, (D).

Antibodies against human α5β1, including volociximab, inhibit angiogenesis and impede tumor growth *in vivo *[21,22]. As volociximab does not cross-react with rodent integrin, these earlier studies were conducted in rabbit and monkey models. We therefore considered whether 339.1 affects tumor growth in syngeneic and xenograft tumor models. In the murine endothelial-derived angiosarcoma model, SVR, 339.1 targets both tumor and tumor vascular endothelial cells. 339.1, administered intraperitoneally to mice bearing established tumors, thrice weekly at 10 mg/kg, inhibited tumor growth by approximately 60% (*p *= 0.007; Figure 6A). In the A673 human xenograft model, 339.1 targets vascular endothelial cells, but not tumor cells. 339.1 inhibited tumor growth in these models by approximately 40% relative to tumors from control-treated mice (*p *= 0.022; Figure 6B). As in the C32 and MDA-MB-231 xenografts, vessels stained positive for integrin with 339.1 in both of these xenograft models (data not shown). Sections from A673 tumors treated with 339.1 were assessed for vessel density by immunohistochemistry using an antibody against anti-CD31 (Figure 6C). Vascular density was significantly decreased in tumors from treated animals compared to tumors that received vehicle (*p* = 0.011; Figure 6D); statistically significant results were obtained in a second experiment employing 25 mg/kg 339.1 three times weekly (15 animals per group; 47% inhibition, p < 0.001), whereas two additional experiments utilizing the 25 mg/kg dosing schedule did not show significant effects (5 animals per group each; 1%, p = 0.96 and 10%, p = 0.82). These results confirm that 339.1, like volociximab, impedes tumor growth, at least in part, by inhibiting angiogenesis.

## Discussion

The strategy of targeting angiogenesis to inhibit cancer progression has received increasing attention in recent years. Despite the recent approval of targeted therapies in this area, optimizing the use of anti-angiogenic drugs in the clinic has been difficult. Challenges that face anti-angiogenic agents that are currently under development include choosing disease areas that might benefit most, optimizing combination strategies with existing standards of care and defining patient populations that might respond best to therapy. Preclinical models of disease provide the best opportunity for addressing these issues, therefore appropriate reagents for use in these systems are essential for driving drugs through development.

Volociximab has been shown to inhibit the growth of new blood vessels in preclinical models of ocular angiogenesis [21]. This effect was found to translate into decreased tumor growth in the rabbit VX2 carcinoma model [22]. These experiments provided a strong proof of concept demonstration of volociximab activity *in vivo *and defined a novel mechanism of action for angiogenesis inhibition. However, the VX2 model is limited in that it represents a very aggressive tumor, must be passaged *in vivo*, is carried out in immunocompetent animals (resulting in antibody clearance) and requires large amounts of antibody. To further define volociximab mechanism of action and identify appropriate settings for its use in tractable animal models of cancer, it was therefore imperative a similar reagent with activity in mouse be generated.

A number of antibodies against mouse α5β1 are available commercially. We have found that although some of these antibodies inhibit binding of α5β1 to fibronectin, none inhibited other biological functions, such as migration, *in vitro *angiogenesis or tumor growth *in vivo *(unpublished observations; [23,24]). However, the α5 knockout mouse is embryonically lethal due to gross defects in vascular architecture [28], suggesting that in mice, as in humans, α5β1 is important for blood vessel formation and/or integrity. The new panel of reagents described herein represents a number of α5- and β1-specific antibodies. Of note, Fc-fusion protein-based immunizations resulted in a higher proportion of α5-specific antibodies, whereas placenta-based immunization resulted in a higher proportion of heterodimer-specific antibodies, including 339.1 (data not shown). As the overall number of antibodies produced by each method was similar, this suggests that the purified material may have resulted in similar immunogenicity while maintaining a more native quaternary structure *in vivo*. In either case, many of the antibodies that bound α5 or were specific for α5β1 heterodimer blocked binding to fibronectin and competed, at least in part, with one another in ELISA or FACS assays (data not shown). Of these antibodies, one group cross-reacted with human integrin, while another did not, suggesting that at least two distinct epitopes were represented. This implies that inhibition of binding to fibronectin can be achieved through blocking at multiple sites, possibly through steric hindrance. Importantly, not all antibodies that block binding to fibronectin have equivalent biological function *in vitro *or *in vivo*. 517-2 and 339.1, for example, each bind with high affinity (0.21 nM and 0.59 nM, respectively) block binding to fibronectin and inhibit migration. Moreover, both antibodies have rat IgG1 constant regions, which like volociximab, a human IgG_4_, would be predicted to lack significant effector activity. However, only 339.1, which does not cross-react with human α5β1, elicits significant cell death *in vitro *and inhibits angiogenesis and tumor progression *in vivo*. This finding suggests that although these antibodies have similar biological functionality and similar affinities, initiation of the cell death program requires binding to a highly specific epitope. This result also suggests that 339.1 binds to the murine cognate of the epitope recognized by volociximab, which would be predicted to be non-homologous between mouse and human α5β1, since volociximab does not cross-react with mouse integrin. A corollary of this hypothesis is that an antibody that recognizes both human and mouse integrin would not bind this important epitope, and therefore might not elicit cell death, as is the case with 517-2.

339.1 inhibits tumor growth in an A673 rhabdosarcoma model. This model was chosen to evaluate anti-α5β1 activity because it was reported to be sensitive to the mouse parent antibody of bevacizumab, A.4.6.1, suggesting that its growth is highly dependent on angiogenesis [8]. However, A.4.6.1 and bevacizumab do not inhibit tumor growth in other xenograft models to the same extent as in the A673 model [29,30]. The reasons for this are not fully understood; 339.1 is currently being evaluated in additional xenograft models to determine if similar differences in sensitivities are observed with this antibody. Comparing xenograft models that respond to 339.1 to varying degrees may reveal molecular mechanisms that will help stratify patients and illuminate combination strategies that might best slow disease progression. In addition, the combination of 339.1 and volociximab is currently being assessed in xenograft models to determine the effect of targeting both tumor and host α5β1 *in vivo*.

## Conclusion

The results described in this report represent the first description of a mouse-specific α5β1 inhibitor that blocks EC function, angiogenesis and tumor growth *in vivo*. These results demonstrate that blocking α5β1 function in mouse models results in an inhibition of tumor growth through a decrease in vessel density. The identification and characterization of anti-α5β1 antibody 339.1 will facilitate further exploration of α5β1 function and the utility of integrin blocking strategies in disease.

## Competing interests

All authors are current or former employees of PDL BioPharma, Inc.

## Authors' contributions

VB developed the screening strategy, participated in the design of the xenograft studies, generated antigen, drafted the manuscript and performed the statistical analysis. DZ participated in the design of the screening strategy and carried out all of the *in vitro *experiments. MF participated in the design, coordination and execution of the *in vivo *studies. PS generated the hybridoma lines and performed the initial screening of the clones. MW performed the BIAcore analysis and PEW contributed to the development of the *in vitro *angiogenesis assay. DP and RBD developed the vectors and methodology for recombinant protein generation. DTC oversaw the immunohistochemistry and accompanying quantitative analysis. VR helped to conceive the study, contributed to its design and coordination and helped draft the manuscript.
